# Potential Effects of Music on Non-Motor Symptoms in Parkinson’s Disease: Translating Mechanisms to Therapy

**DOI:** 10.3390/neurolint18030045

**Published:** 2026-02-26

**Authors:** Christopher G. Ballmann, Daphne G. Schmid, Rebecca R. Rogers, Hannah K. Oakes, Shelby C. Osburn

**Affiliations:** 1 Department of Human Studies, University of Alabama at Birmingham, Birmingham, AL 35294, USA; scosburn@uab.edu; 2 Department of Physical Therapy, University of Alabama at Birmingham, Birmingham, AL 35294, USA; 3 Department of Applied Physiology and Kinesiology, University of Florida, Gainesville, FL 32611, USA; daphs03@ufl.edu; 4 Department of Family and Community Medicine, University of Alabama at Birmingham, Birmingham, AL 35294, USA; rrrogers@uab.edu; 5 Rooted Music Therapy, Birmingham, AL 35294, USA; hannah@rootedmusictherapy.com

**Keywords:** Parkinson’s disease, neurodegeneration, music, non-motor

## Abstract

Non-motor symptoms (NMSs) are highly prevalent in Parkinson’s Disease (PD) and contribute significantly to disease severity, progression, and diminished quality of life. NMSs are rooted in both physiological and psychological domains and include emotional dysfunction, autonomic dysregulation, cognitive impairment, pain exacerbation, and neural deficits. While pharmacological approaches are often employed for the alleviation of non-motor symptomology, modest efficacy and adverse side effects may limit their practical utility for individuals with PD, leaving the need for the identification of complementary approaches. Music interventions have emerged as potential adjunctive therapeutic approaches that may positively modulate NMSs in both physiological and psychological domains. Physiologically, music interventions have been shown to alter autonomic activity and pain/sensory perceptions and mediate neurotransmitter release related to arousal, physical effort, and stress. Psychologically, music interventions, both passive and active, have been shown to modulate emotional regulation, motivation, attention, and cognitive performance. Emerging evidence utilizing neuroimaging and behavioral techniques further supports this and suggests music-induced benefits even in the presence of advancing neurodegeneration. Overall, findings from this narrative review suggest music may serve as a potential non-invasive adjunctive therapeutic tool to counteract PD-induced NMSs by adaptively modulating physiological and psychological processes. This narrative review aims to gather current evidence on the physiological and psychological mechanisms underlying the benefits of music and proposes potential therapeutic translation for NMSs in PD. Furthermore, current difficulties, gaps in knowledge, and needs for future research are discussed with the goal of informing directions for clinical translation.

## 1. Introduction

Parkinson’s disease (PD) results in pathological aging underpinned by dopaminergic insufficiency, resulting in motor and non-motor symptoms (NMSs). PD is one of the fastest-growing neurological diagnoses in aging populations globally, with prevalence growing ~2.5 times higher from 1990 to 2016 [1]. While motor dysfunction and impairment are often what prompt a formal clinical diagnosis, NMSs may appear years before motor symptoms begin and are central contributors to disease severity, disability, and worsening of quality of life [2,3]. NMSs describe a broad spectrum of pathological consequences rooted in both physiological and psychological dysfunction, including affective disturbances (e.g., anxiety and depression), autonomic dysregulation, exacerbation of pain, cognitive deficits, apathy, and lack of energy/fatigue [3,4]. Consequently, NMSs may negatively influence functional capacity, completion of daily tasks, and limit mental performance necessary for optimal function. Implementation of pharmacological strategies to manage NMSs is common in the treatment of PD but often suffers from heterogeneity of efficacy and induction of unwanted side effects and may only target a single symptom [5]. Thus, identifying practical adjunct therapies that may aid in overcoming difficulties in current treatment regimens, while encompassing multiple NMS domains concomitantly is vital to optimizing treatment options for people with PD.

The application of music, in both acute and chronic interventions, has emerged as a promising complementary therapeutic approach due to the capacity to adaptively modulate physiological and psychological processes, which may translate to the alleviation of NMSs. Indeed, music potently induces neuromodulatory effects and alters the activity of a variety of neural networks, including paralimbic, limbic, and cortical regions of the brain, which are often involved in the neuropathology of NMSs in PD [6,7,8]. Through the modulation of these pathways, music has been shown to alter autonomic activity, neurotransmitter signaling (e.g., dopamine and norepinephrine), stress reactivity, and pain/fatigue perception [9,10]. Music interventions exert positive effects on mood regulation, physical effort, arousal, and motivation [11]. Furthermore, music interventions have been associated with improved emotional well-being, memory, and cognitive engagement. While precise mechanisms for these benefits are still being elucidated, advanced neuroimaging studies utilizing function magnetic resonance imaging (fMRI) and positron emittance topography (PET) have suggested that music-induced activation of brain circuitry is important for responses to motivation, emotion, and stress, including the nucleus accumbens, amygdala, and various cortical structures (e.g., prefrontal cortex and cingulate cortex), which are known contributors to NMSs in PD [6,7,9]. Thus, there is a firm physiological and psychological rationale for the potential benefits of music interventions in PD, which may aid in counteracting NMSs in particular.

While music has been studied and employed as an adjunct intervention in PD and neurodegeneration across the literature [12], most of the existing work has largely focused on motor symptoms or motor rehabilitation with comparatively less emphasis on NMSs. Furthermore, findings in the context of NMSs are largely fragmented across the literature based on particular disease populations, methodological approaches, and outcome focus [12,13]. A comprehensive review of the available evidence of physiological and psychological underpinnings of music-induced benefits in the context of NMSs is therefore warranted in order to highlight the mechanistic understanding and therapeutic potential of music interventions for people with PD. This may also aid in the facilitation and advancement of future scientific inquiry and clinical application. While contextual and methodological (e.g., music characteristics) factors are known to influence responses to music, thus contributing to widespread heterogeneity of findings in the literature, the differentiation of this is beyond the scope of the narrative review and encompasses both passive and active music therapies as a general adjunct termed “music interventions”. Accordingly, the purpose of this narrative review is to synthesize the available evidence on the mechanisms underpinning the physiological and psychological benefits of music interventions and how this may theoretically aid in counteracting PD-induced NMSs as an adjunctive therapy. This narrative review also highlights current challenges, gaps in scientific knowledge, and potential for therapeutic application aimed at optimizing music-based interventions to improve NMSs in PD.

## 2. Methods

### 2.1. Design and Purpose of the Narrative Review

This narrative review sought to summarize and synthesize the available literature from multiple disciplines on the physiological and psychological effects of music interventions in order to offer new perspectives on the therapeutic potential of music for NMSs in PD. This work strategically integrates findings from fields of neuroscience, music medicine, music therapy, psychology, and physiology into a narrative approach to allow for a wider integration of mechanistic, behavioral, and therapeutic findings that may otherwise be difficult to encompass through systematic or meta-analytic approaches. The included body of literature concentrates on identifying key studies that clarify mechanisms of action, demonstrate therapeutic relevance to PD, and establish theoretical foundations for understanding the potential benefits of music interventions on NMSs. By synthesizing evidence across diverse populations to highlight mechanistic findings and application to relevant PD-induced NMSs, we aim to bridge the gap between theory and practice and suggest future directions for applying music interventions in PD.

### 2.2. Literature Search, Inclusion, and Synthesis

The literature search was conducted utilizing various online databases including PubMed/MEDLINE, Google Scholar, and Scopus. Peer-reviewed publications from the past 20 years (2005–2025) were included in attempts to simultaneously encompass foundational mechanistic evidence of music with the latest progress of clinical knowledge. Only evidence that was available in the English language was included. In order to capture pertinent evidence pertaining to PD-induced NMSs, the mechanistic underpinnings of music, and therapeutic relevancy, various search terms were used, including Medical Subject Headings (MeSH), entry terms, and applicable keywords, which are categorized in Table 1. Combinations of terms were included with “AND” and “OR” to aid in narrowing the search results. Primarily, human experimental or clinical trials were included, with some trials involving animals if mechanistically relevant to PD or music interventions. Relevant systematic and meta-analytic reviews were also included to bolster the scope of evidence. All case reports/series, abstracts, conference proceedings, and non-peer-reviewed sources were not considered for inclusion. Strength of evidence for each piece of literature was qualitatively and descriptively assessed by at least two authors, then separated into themes/categories.

## 3. Physiological and Psychological Effects of Music

### 3.1. Overview

**Figure 1:**
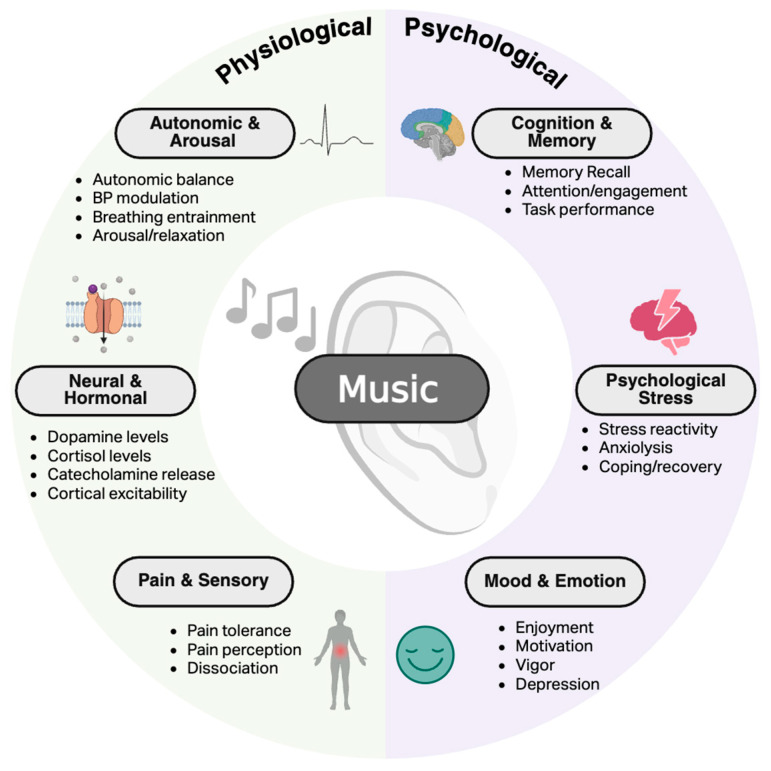
Overview of the impacts of music on physiological and psychological outcomes relevant to non-motor processes. Created in BioRender. Ballmann, C. (2026) https://BioRender.com/jkbovzv (accessed on 20 February 2026).

The effects of music on the physiological and psychological underpinnings of non-motor processes have been widely established in healthy adults and across various populations (Figure 1). The purpose of this section is twofold: (1) to provide a brief overview of foundational knowledge related to how music interventions alter physiological and psychological mechanisms controlling non-motor processes; (2) to briefly summarize current knowledge of the benefits of music interventions to establish a framework for the potential translation to clinical populations experiencing non-motor system dysfunction, namely PD. For a more generalized review of the potential benefits of music interventions in neurological conditions, refer to [10].

### 3.2. Physiological Domain

Music interventions have been shown to induce systemic changes largely mediated through neurophysiological mechanisms that enhance brain function and reactivity. Downstream of this, complex coordination of potent changes to autonomic regulation, neural/hormonal responses, and alterations to nociceptive stimuli underpin the collective physiological benefits of music [14]. Importantly, physiological changes induced by music are highly relevant to the enhancement or function of non-motor processes in PD and other neurodegenerative diseases. While mechanistic knowledge of how music influences neurophysiological processes remains incomplete, the current understanding provides a foundational rationale for using music interventions to strategically alter non-motor processes, especially relevant to NMSs in PD.

#### 3.2.1. Autonomic Activity and Arousal

Autonomic responses and arousal are largely mediated by the balance of sympathetic and parasympathetic activation under control by brainstem and midbrain structures that collectively orchestrate systemic and visceral effects. Dysregulation of the autonomic nervous system (ANS) results in both physiological and psychophysiological consequences, including blood pressure irregularities, hemodynamic alterations, severe fatigue, and poor adaptivity to stress [15,16]. Music interventions have been shown to alter ANS activity, particularly cardioautonomic, through a complex coordination of various brain regions, including the hypothalamus, ventral tegmental area, nucleus accumbens, and insula [17,18]. Across studies, music has been reliably shown to alter cardioautonomic outcomes, including heart rate, heart rate variability, and blood pressure [19]. Furthermore, music interventions may increase autonomic arousal and electrodermal activity, which is thought to be primarily mediated by transient changes in sympathetic activity, which are highly linked to emotion [20,21]. Further supporting this, music has been shown to result in psychophysiologic changes via a “psyching up” effect, thereby enhancing arousal and feelings of energy [11,22,23]. However, it should be noted that modulation of ANS activity and arousal are particularly dependent on the form or characteristics of music. For example, high-frequency/tempo music tends to lead to increases in sympathetic responses (i.e., increased heart rate and blood pressure), while low-frequency/tempo music favors parasympathetic activation [24,25]. Furthermore, varying music genres have been suggested to alter heart rate variability and autonomic balance [26]. Thus, while music has been widely shown to alter ANS activation and arousal through physiological processes, the characteristics and selection of music are highly context-dependent and explain heterogeneous responses based on methodological approaches.

#### 3.2.2. Neural and Hormonal

Neurophysiological effects of music interventions have been described to be driven through the interaction of neurohormonal regulated processes that ultimately influence emotional regulation, motivated behavior, stress responses, and autonomic reactivity [7,17,27]. Indeed, music and auditory stimuli have been suggested to activate auditory cortex-mediated signaling cascades throughout the brain, including limbic (e.g., nucleus accumbens, hypothalamus, amygdala) and frontal (e.g., cingulate cortex and prefrontal cortex) regions [7,28]. Key neurotransmitters, including dopamine, serotonin, and norepinephrine, have been shown to be sensitive to music, which, when coordinated, may result in systemic changes to behavioral, perceptual, and psychophysiological responses [29]. Music-induced changes in dopaminergic and serotonergic signaling have been purported to alter reward processing, motivational behavior, emotional regulation, and physical effort allocation [6,7,8,30]. Additionally, some evidence has suggested music interventions exert their effects through the modulation of endogenous opioid and GABAergic systems, contributing to altered activity in cortical and subcortical brain regions, thereby modulating neurotransmitter-mediated changes in mood, pain, and feelings of anxiousness [31,32]. Music interventions have also been shown to improve immune function and promote neuroplasticity through the modulation of dopamine release and stress reduction [6,33,34], though some of these benefits may be specific to people who have higher musical aptitude [33]. Use of music with the intent for relaxation or the reduction in stress may modulate signaling of the hypothalamic–pituitary–adrenal axis, specifically resulting in lower cortisol levels [35]. On the contrary, motivational or stimulative music has been suggested to upregulate catecholamine release (e.g., epinephrine and norepinephrine) [36,37]. Overall, music interventions induce neurochemical changes centrally and peripherally that ultimately result in systemic physiologic changes that may result in stimulative or relaxing effects depending on the context.

#### 3.2.3. Pain and Sensory

While the benefits of music interventions on pain management are widely documented in various conditions [38,39], the physiological mechanisms that underlie these effects are not fully understood. There are two primary hypotheses that have been suggested to explain possible physiological underpinnings of hypoalgesic effects from music interventions: (1) dopaminergic-mediated changes in reward/pleasure processing and (2) modulation of endogenous opioid signaling. Music increases network activity of cortical and subcortical brain regions, resulting in music-dependent alterations of dopaminergic signaling attributed to increases in subjective enjoyment, pleasure, and reward valuation [40,41]. Dopaminergic signaling may also alter descending pain inhibitory pathways and affective responses, thereby resulting in hypoalgesic effects, although this has not been fully elucidated in the context of music [42]. In addition to dopamine, changes in opioid signaling within limbic and paralimbic brain regions may also explain music-induced changes in pain perception. Pharmacologic blockade of opioid pathways (e.g., naltrexone) has been shown to blunt musically induced feelings of pleasure and likely modifies music-induced hypoalgesia [43]. Furthermore, changes in descending pain modulatory circuits, which are heavily influenced by opioid activity, have been suggested to be modulated by music [39,44]. This has been further supported by neuroimaging evidence showing music blunts descending pain signaling in the spinothalamic tract, although the contribution of endogenous opioids to this is not fully clear [45]. Recent evidence using PET imaging has shown that music induces endogenous opioid activity, specifically on μ-opioid receptors, in cortical and subcortical regions that were associated with pleasurable experiences of music [31]. Importantly, physiological mechanisms underlying neurochemical changes from music may also alter attention and cognitive processing, leading to psychophysiological states of dissociation, thereby enhancing hypoalgesia. Music interventions have been well established to alter attentional focus, discomfort, and perceptions of exertion, which may, at least in part, be mediated by the aforementioned mechanisms [45,46,47,48]. Thus, pain-modulating effects of music interventions likely result from a host of coordinated factors that together are mediated by changes in reward, descending pain signals, and dissociation, which may be targeted strategically to decrease pain.

### 3.3. Psychological Domain

Beyond well-established physiological impacts, music has significant psychological effects, supporting its therapeutic potential in improving symptomology across various diseases comprehensively. Music’s psychological influence arises from complex interactions among cognitive, emotional, and motivational systems, producing effects that extend beyond auditory processing [49]. Potent psychological benefits of music have been widely reported, including alterations in mood regulation [50], attention [51], memory consolidation [52], stress responses [53], and motivation [54,55]. Importantly, these effects have high relevance to neurodegenerative diseases like PD, which support the potential of therapeutic translation. Understanding common links between music interventions and cognitive/behavioral psychological processes provides a foundation for exploring how music interventions could help address cognitive decline, emotional dysregulation, anxiety, apathy, and other NMSs experienced by people with PD.

#### 3.3.1. Cognition and Memory

Cognition and memory represent a complex set of neural processes of the acquisition, processing, and storage of assimilated experiences by which behavior is guided [56]. Various inputs from external factors, including auditory stimuli such as music, deeply mediate cognitive processes. Music interventions have been shown to alter activation and functional connectivity of a myriad of brain regions important for cognitive function and memory, including the hippocampus, basal ganglia, and frontal/temporal cortices [8]. Consequently, music interventions have been suggested to alter memory encoding, retrieval of stored experiences, and various aspects of executive functioning through both global and regional cascades of neural activation [8,57]. For example, music interventions have been shown to improve global cognition while also improving more distinct cognitive aspects such as functional memory and executive function [57]. At the attentional filtering stage, salient auditory properties of music can enhance selective attention by giving rhythmic cues that guide focus toward relevant information while masking distracting environmental noise [51,58]. Music processing also engages overlapping neural pathways that support executive functioning networks, which may contribute to enhanced inhibition, working memory, or task switching [59]. Memory encoding, consolidation, and retrieval have been suggested to be supported through rhythmic–melodic patterns of music that provide temporal cues for information chunking, sequencing, and long-term memory retrieval [60]. By engaging multiple stages of information processing simultaneously, from attentional filtering to memory consolidation, music-based interventions may offer a comprehensive approach to supporting cognitive function.

#### 3.3.2. Mood and Emotion

Mood and emotional regulation are mediated largely through coordinated activity of various brain regions responsible for governing perceived reward, motivation, psychological arousal, and affect [61]. Indeed, the dysfunction of various brain structures (e.g., nucleus accumbens, amygdala, cingulate cortex) within mesolimbic, mesocortical, and paralimbic pathways has been implicated in a number of mood disorders and neuropsychiatric symptoms [61,62]. Music interventions have been repeatedly shown to alter activation and connectivity of these emotional circuits and pathways in the brain, thus controlling various facets of mood and emotion [8]. Neuromodulatory properties of music have been attributed to widespread changes to neurotransmitter release in the brain, including dopamine, serotonin, and norepinephrine [8,29]. Importantly, music-induced neurophysiological changes have been widely shown to result in improvements in subjective emotional measures, including vigor, arousal, enjoyment, and overall feelings of well-being [11,63]. Furthermore, music can support emotional processing and affect regulation facilitated by cognitive reappraisal, suppression, and altered behavioral responses to stimuli [64]. This is supported by evidence showing that music may be used to induce attentional redirection and mood transition by which the listener may be able to choose certain characteristics of music to achieve a desired affective state (i.e., transition from feelings of sadness to enjoyment) [65]. Supporting this further, some forms of music have been shown to be effective at inducing a “psyching up” effect, thereby enhancing psychological arousal, vigor, and feelings of energy, particularly in situations where high amounts of effort allocation are necessary [11,22,23]. Music may also alter the motivational state, support goal-directed behavior, and enhance feelings of enjoyment by activating reward pathways and providing positive emotional experiences [50]. Taken together, the converging evidence underscores how music can simultaneously activate reward-related motivational schemas, alter emotional responses, and reinforce higher-order executive regulations, thereby enhancing mood across multiple psychological domains.

#### 3.3.3. Psychological Stress

Similarly, mechanisms that mediate mood and emotion regulation, such as the amygdala, prefrontal cortex, and cingulate cortices, have also been implicated in responses and adaptive control of psychological stress [66,67]. In particular, these structures have been implicated in emotional processing, threat detection, and global stress regulation. This is further confirmed by neuroimaging evidence showing an induction of psychological stress results in activation of the anterior cingulate cortex, with hypoactivation of limbic structures [68]. Music is a potent mitigator of psychological stress through the engagement of both stress-regulating and coping processes. Stress-reducing effects of music interventions partially manifest through altering appraisal and emotional responses to environmental factors, including social and contextual stimuli [69]. This is likely related to the regulation of arousal levels, resulting in increased positive and decreased negative feelings, subjective worry, and state anxiety [70,71]. Music interventions may also alter emotional valence under stressful conditions, whereby the listeners subjectively perceive more “positive” or pleasant feelings, thereby allowing for greater adaptability [72]. Anxiolytic effects of music have similarly been well described across various populations. Indeed, music-induced anxiolysis has been attributed to increased dissociation from worry or rumination, enhancements of state mindfulness, and promotion of positive affect [35,73]. Music may further improve resilience to psychological stress through enhancement of coping and recovery by down-regulation of negative affect, reducing cognitive burden through dissociation, self-efficacy enhancement, and stabilization of mood [46,74,75]. Collectively, the psychological mechanisms underlying the therapeutic effects of music interventions span attentional redirection, cognitive reappraisal, emotional regulation, and reward system activation, demonstrating multi-faceted benefits as a strategy to combat psychological stress.

## 4. Therapeutic Potential on Non-Motor Symptoms in Parkinson’s Disease

### 4.1. Overview

**Figure 2:**
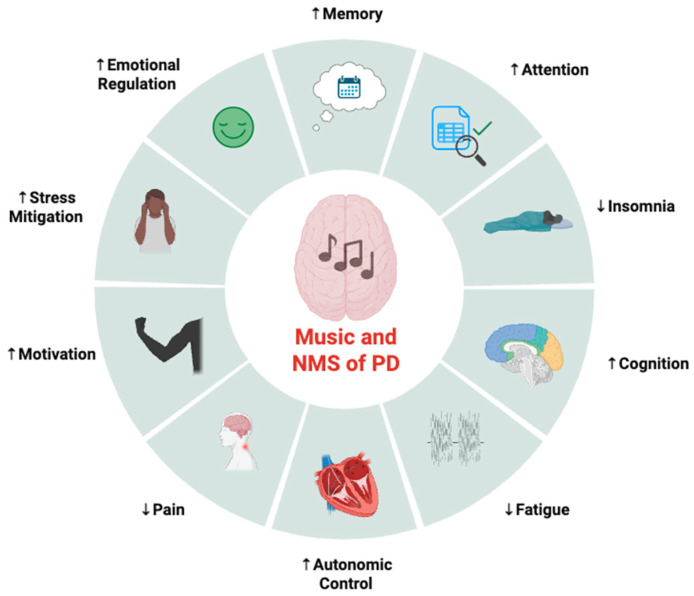
Potential areas of therapeutic benefit for music interventions on non-motor symptoms (NMSs) of Parkinson’s disease (PD). Created in BioRender. Ballmann, C. (2026) https://BioRender.com/ck569q3 (accessed on 20 February 2026).

To date, the majority of evidence available suggesting therapeutic benefits of music in PD are focused on motor symptoms and processes, leaving the potential impact on NMSs of PD relatively unknown. Thus, the purpose of this section is to provide a theoretical and mechanistic rationale across various populations of the potential benefits of music interventions in the context of PD-induced NMSs (Figure 2). While the translation of proposed mechanisms to therapy for NMSs in PD is largely speculative at this time, the following section may serve as a framework for future clinical and experimental trial exploration into how music may be used as a non-invasive, feasible, and enjoyable adjunct therapy to improve NMSs in PD.

### 4.2. Cognition and Executive Function

Cognitive decline in PD is driven by the convergence of multiple degenerative processes, including Lewy body accumulation, dysfunction of neurotransmitter systems, and co-occurring pathologies such as cerebrovascular disease and chronic inflammation [76]. PD affects cognition across multiple domains, and meta-analytic evidence shows substantial cross-sectional differences between cognitively normal PD patients and those with mild cognitive impairment (PD-MCI) across multiple neuropsychological measures [77]. Dopamine receptor dysfunction contributes to this decline by disrupting higher-order brain functions, including decision-making, attention, and behavioral flexibility. Dopamine depletion impairs receptor-mediated activation distributed across brain regions critical for cognition and is widespread (Table 2). This dysfunction disrupts the cortico-basal ganglia circuit, compromising striatal–prefrontal communication and resulting in deficits in executive function, working memory, cognitive flexibility, and attention [78,79]. PD also involves degeneration of the noradrenergic locus coeruleus and basal forebrain cholinergic system, neuromodulatory networks which are essential for sensory detection, attention, executive function, and memory [80,81]. Considering the significant predictive power of declining executive function, therapeutic methods aimed at maintaining or improving it could be essential in delaying the worsening of cognitive symptoms in people with PD-MCI [76].

**Table 2:** **Dopamine receptor subtypes and cognitive consequences of depletion as observed in PD.** This table compiles findings from [76,82,83,84,85].

Dopamine Receptor	Associated Brain Regions	Cognitive Implications When Depleted
D1	striatum, substantia nigra pars compacta, nucleus accumbens, hippocampus, anterior cingulate cortex	↓ executive functioning, ↓ motivation, ↓ attention, ↓ long-term memory, ↓ cognitive flexibility, ↓ learning
D2	Striatum, hippocampus substantia nigra, insular lobe, prefrontal cortex, anterior cingulate cortex	↓ executive functioning; ↓ working memory; ↓ inhibition, ↓ error detection
D3	Nucleus accumbens, insular cortex, amygdala, hypothalamus	↓ inhibition, ↓ cognitive flexibility, ↓ executive functioning
D4	Frontal cortex, amygdala, striatum	↓ attention, ↑ hyperactivity, ↓ executive functioning
D5	Hippocampus, thalamus, striatum, nucleus accumbens	↓ long-term memory, ↓ working memory, ↓ cognitive regulation, ↓ learning

Few studies have directly assessed the effects of music on general cognitive function in PD. This evidence gap is notable, as neurophysiological findings suggest music interventions may support prefrontal–parietal networks involved in cognitive processing [86]. However, some emerging evidence has suggested that music interventions, when integrated into an eight-week computer-assisted PD rehabilitation program, result in significantly greater cognitive gains than those receiving identical rehabilitation without musical augmentation [87]. Specifically, the addition of music to the rehabilitation program resulted in greater improvements across global cognition, executive function, inhibitory control, processing speed, and attention test, whereas controls improved only in inhibitory control. Additionally, when executive function was examined in isolation, music integrated into therapeutic strategies showed benefits for cognitive flexibility and inhibition [88,89]. Furthermore, the rhythmicity of music is thought to support cortico-thalamocortical and basal ganglia–thalamocortical network communication, both of which are impaired in PD and critical for attentional orientation and information processing [90,91,92]. This sensory–motor integration is theorized to redirect neural processing and bolster executive functioning [93]. Evidence for these ideas is evident from P300 event-related potential activity recorded through electroencephalography, indicating improved attention allocation, updates in working memory, and overall cognitive processing efficiency [94]. P300 modulations translate into measurable cognitive improvements, including sustained and selective attention, faster processing speed, enhanced working memory, improved executive function, and inhibitory control. In PD, where dopaminergic deficits impair attention and executive function, music-induced P300 enhancement may reflect compensatory neural mechanisms that support cognitive rehabilitation, particularly when rhythmic cues engage intact motor-timing networks to bypass compromised basal ganglia circuits [94,95]. Despite these promising preliminary findings, larger randomized controlled trials are needed to establish optimal intervention parameters, clarify underlying neural mechanisms, and determine whether music-based approaches can delay cognitive decline in PD.

### 4.3. Memory and Attention

PD results in progressive declines in cognitive function, which are largely underpinned by dopaminergic dysfunction and Lewy body aggregates in key brain regions involved in learning, memory, and executive functions [96]. These pathological processes converge to impair various neurotransmitter systems and neural circuits essential for memory encoding, consolidation, and retrieval. Based on longitudinal data from newly diagnosed PD patients, dementia prevalence reaches 83% among 20-year survivors, suggesting a substantial cumulative risk of cognitive decline [97]. The development of Parkinson’s disease dementia (PDD) correlates with increasing age and appears to result from an interplay of multiple neuropathological processes rather than a single underlying mechanism. Dopaminergic terminal denervation in the associative dorsal caudate nucleus and nucleus accumbens is linked to PDD development [98,99]. Caudate denervation affects cortico-basal ganglia circuits, impairing communication between the striatum and prefrontal cortex. Consequently, deficits in working memory, attention, cognitive flexibility, and visuospatial skills, all of which depend on cortical dopamine regulation, are observed in PD [100]. Additionally, locus coeruleus neurons that produce norepinephrine degenerate early in PD, impairing memory-encoding processes that rely on frontal cortex–hippocampal interactions [76,101]. Neuromelanin-sensitive MRI detects a reduced locus coeruleus signal even in PD-MCI, with further decline correlating with worsening cognitive function in PDD [102]. This degeneration significantly contributes to the attentional deficits seen in PD-MCI and PDD, including impaired sensory signal detection, arousal, attentional orientation, behavioral flexibility, and working memory. As attention and arousal are essential for effective memory encoding, the loss of norepinephrine may hinder memory formation. Furthermore, neurodegeneration in brain regions such as the hippocampus is linked to declines in overall cognitive performance, including long-term, spatial, and working memory, as evidenced by decreasing bilateral hippocampal volume [103,104].

Although few clinical trials have directly examined the relationship between music interventions and memory in PD, research in other neurodegenerative diseases suggests that music may be an effective therapy for protecting against memory decline or as a tool to create musical memories that are often maintained even as diseases progress significantly [105,106]. Music listening engages the mesocorticolimbic pathway, amplifying dopaminergic activity, particularly in the nucleus accumbens [7,8]. As PD impairs the cortico-basal ganglia circuits that underpin memory consolidation, motivation, and reward, music may act as a compensatory stimulus, stimulating dopaminergic release in the nucleus accumbens [8]. This, in turn, projects to the anterior cingulate cortex and downstream to hippocampal circuits, thereby counteracting dopaminergic deficits, orienting attention, enhancing motivation, and possibly supporting memory encoding, consolidation, and retrieval in people with PD. However, the strength and consistency of music-induced enhancements in long-term memory have not yet been demonstrated to the same extent as its reported benefits for executive functioning in PD [93,107]. Future longitudinal studies and clinical trials will be needed to determine to what extent music interventions can attenuate or mitigate memory and attentional loss in PD-MCI and PDD.

### 4.4. Insomnia and Sleep

Sleep–wake disturbances are widely experienced NMSs for people with PD, with estimates of prevalence ranging from 30 to 80% [108]. While dependent on the clinical sampling and diagnostic criteria used, sleep disturbances may manifest in excessive fatigue, daytime sleepiness, restless leg syndrome, and/or alterations in rapid-eye movement (REM) sleep behavior [108,109]. Insomnia and PD-associated sleep disturbances often begin early in disease progression and progressively intensify over time [110]. Neurophysiological underpinnings of sleep disturbances have been attributed to multiple mechanisms that often stem from progressive neurodegeneration. Dopaminergic degeneration of pathways involving the basal ganglia has been suggested to alter pathways controlling arousal, which may result in increased nocturnal waking episodes [111]. Furthermore, circadian dysfunction of serotonergic (e.g., raphe nuclei)- and noradrenergic (e.g., locus coeruleus)-dependent structures has been suggested to worsen sleep latency, maintenance, and REM cycling [112]. While pharmacologic interventions have been shown to have efficacy in PD-associated sleep disturbances [113], it is important to note that sleep aids may result in residual drowsiness and may incompletely address the aforementioned neurotransmitter targets [114].

Although randomized clinical trials investigating the effects of music interventions on sleep-related disturbances are sparse, there is substantial evidence in healthy younger and older populations and those experiencing insomnia. Music interventions have been shown to reduce sleep onset latency and may improve sleep efficiency [115]. Furthermore, music interventions may improve global sleep quality and increase total sleep time [116]. It is worth noting that many trials have used passive music interventions whereby relaxing or self-selected music listening prior to sleep produces clinically meaningful outcomes, while less efficacy has been suggested with active music interventions [117]. Mechanistically, music interventions such as this likely modify sleep outcomes through the neuromodulation of autonomic and affective regulating pathways that interact with sleep–wake signaling. Music interventions that are relaxing in nature may result in favorable changes in the activation of autonomic, limbic, and cortical circuits [8]. In turn, this may result in reduced markers of sympathetic drive (i.e., lower heart rate and blood pressure), enhance vagal tone, and extinguish maladaptive stress responses [118,119]. Moreover, sleep disturbances from PD-induced anxiety and depression may be affected by improving emotional regulation and inducing dissociation. At this time, it is difficult to discern if music interventions are primarily associated with reductions in neuropsychiatric outcomes leading to potential improvements in sleep or vice versa, leaving the need for more targeted approaches and manipulation of music intervention characteristics.

### 4.5. Apathy and Motivation Loss

Apathy, or core loss of motivation, is a common non-motor symptom of PD that reduces initiative, effort allocation, and goal-directed behavior. Clinically, this may manifest in reduced interest in activities of daily living, inappropriate emotional responses, and increased difficulty in completing tasks which were previously perceived as valuable or meaningful. While the presentation of apathy in PD may be heterogeneous or vary in intensity, core losses in motivation associated with PD-induced apathy often cause deleterious effects to daily function and quality of life. Apathy has been suggested to occur early in PD progression and affects roughly 40% of all patients, although diagnosis is often complicated by the co-occurrence of other neuropsychiatric conditions such as depression and cognitive deficits [120,121]. Precise causes of PD-induced apathy are not fully clear but are suggested to be primarily related to mesocorticolimbic dopaminergic system dysfunction [122]. The mesocorticolimbic system, composed of structures forming the brain’s primary reward control center, includes the nucleus accumbens and neural projections to cortical brain regions such as the anterior cingulate cortex, which mediate motivated behavior and action. Indeed, mesocorticolimbic dopaminergic system dysfunction has been implicated in PD-associated apathy, including reduced activity of the nucleus accumbens, which regulates reward-seeking and motivated behavior [123]. Structural and functional decrements of the nucleus accumbens and anterior cingulate cortex begin occurring in early-stages of PD and correlate with higher severity of apathy and motivational loss [124,125,126]. Furthermore, PD-induced alterations of connectivity between the nucleus accumbens and anterior cingulate cortex have been implicated in behavioral changes and motivational deficits, which may worsen over time [120,121].

Music may be an effective non-pharmacologic adjunctive therapy for combatting apathy and motivational loss due to PD. Music has been shown to potently alter the activation of mesocorticolimbic pathway structures (e.g., nucleus accumbens and anterior cingulate cortex) and enhances dopaminergic activity in healthy adults [6,7,28]. Importantly, these music-induced changes in mesocorticolimbic activity appear to correspond to similar dysfunctional structures underlying PD-induced apathy. Music listening stimulates the auditory cortex, which is responsible for the perception of auditory/psychoacoustic stimuli, thereby directly or indirectly altering dopaminergic activity of the nucleus accumbens, which dictates music-induced dopaminergic reward experiences and motivational value [7,28]. Music-induced dopaminergic activity from the nucleus accumbens projects to the anterior cingulate cortex, thereby mobilizing motivational behavior and effort allocation [127,128,129]. Music may also induce feedback loops from the anterior cingulate cortex to the nucleus accumbens, perpetuating dopamine signaling, further driving enhanced motivational behavior [128,129]. Furthermore, potentiation of dopaminergic feedback loops between the nucleus accumbens and auditory cortex may further strengthen music-induced reward perception and the motivational state [6,7,28]. Importantly, these music-induced changes in mesocorticolimbic activity appear to correspond to similar dysfunctional structures underlying PD-induced apathy. Bolstering this, music has been widely reported to potently increase feelings of motivation, especially when the music is self-selected or preferred by the listener [10,11]. While clinical confirmation of the aforementioned mechanisms is needed, the neuromechanistic rationale for listening to music may be an innovative means of counteracting apathy and motivational deficits in PD.

### 4.6. Mood and Emotional Dysregulation

Changes in mood and emotional regulation contribute to a myriad of underlying affect-associated NMSs in PD. For example, the presence of PD-associated depression has been estimated to occur in nearly 40% of people with PD and is commonly presented with other comorbid neuropsychiatric symptoms such as anxiety and apathy [130]. Further manifestations of mood dysregulation in PD include anhedonia, irritability, and abnormal emotional reactivity, which are further attributed to worsened disease progression and quality of life [130]. Thus, mood and emotional dysregulation are often considered core features of the pathology of PD, although differences in assessment and clinical thresholds across evidence may complicate precise diagnosis and treatment plans [131]. Underlying causes of emotional dysfunction in PD are multi-faceted and likely occur as a result of broad-level neurotransmitter and neurocircuit dysfunction due to neurodegenerative processes affecting dopaminergic, serotonergic, and noradrenergic systems [122,132,133]. This is further bolstered by evidence that the removal of dopaminergic replacement therapies (e.g., “off” state) results in partial worsening of mood and reward processing [134]. Key affective regulating neurocircuits, including mesolimbic, mesocortical, paralimbic, and prefrontal networks, have also been implicated in PD-associated neuropsychiatric NMSs [133,135,136]. Currently, pharmacologic modulators such as dopamine agonists and selective serotonin/serotonin–norepinephrine reuptake inhibitors are among the first-line therapeutic strategies to improve mood symptoms in PD [137,138]. However, responses to treatment have been suggested to be highly variable based on drug class and tolerability of long-term therapy, which may differ between patients [139].

Given the potential limitations associated with pharmacologic therapies targeting PD-induced mood dysfunction in their totality, complementary approaches such as using music interventions to mediate mood and emotional regulation have a high therapeutic upside. As previously mentioned, music alters activity and connectivity of various brain regions, including limbic, paralimbic, and cortical neural pathways, which together regulate pleasure, reward processing, and emotional regulation. Indeed, music interventions have been widely shown to improve mood, affect, enjoyment, and emotional processing [11,23]. Psychophysiological benefits of music interventions have been shown across various populations and health statuses, including individuals with PD and neurodegeneration [10,140]. Furthermore, music may be an effective adjunct therapy to rehabilitation practices for PD, as the addition of music to physical therapy programs has shown improvements in emotional well-being and quality of life in PD patients [141]. Mechanistically, the auditory cortex directly interacts with various structures regulating affect, including the ventral tegmental area, amygdala, prefrontal cortex, and nucleus accumbens [41,142,143,144]. As previously mentioned, music has also been shown to modulate the neurotransmitter release of dopamine, serotonin, and/or norepinephrine, which are highly integral to proper mood and emotion control [29]. Importantly, many of these neural pathways affected overlap with those of dysfunctional neural pathways, which are thought to mediate PD-associated mood disorders and emotional dysfunction, suggesting high potential for therapeutic translation. Although largely speculative at this time, the similarities in affected neural pathways suggest that music interventions may provide partial compensation for PD-induced mood and emotional dysfunction, allowing for non-invasive adjunct therapy to improve the mood state and enhance positive affect. Additional PD-specific clinical and experimental trials focused on altering emotional and affective pathways are needed to understand potential acute and chronic benefits of using music as an adjunctive therapy to combat mood dysregulation as an NMS.

### 4.7. Anxiety and Stress

Anxiety and heightened stress levels are commonly reported by individuals with PD and have been suggested to impair functional ability and quality of life and may further exacerbate overall disease progression and prognosis. Phenotypically, PD-induced anxiety disorders may be generalized or specific (i.e., social and phobia-based), with prevalence estimated to occur in roughly 31% of PD patients [145]. Clinical presentation of anxiety symptoms in PD is highly variable between individuals but includes persistent worry, panic episodes, behavioral changes of avoidance, and somatic changes including headache, hyperventilation, or muscle tension [146]. Coexistence of anxiety with other neuropsychiatric symptoms (e.g., depression) is common, and the intensity of clinical symptoms has been shown to be correlated with duration and severity of disease [146]. Causes of PD-induced anxiety are uncertain but are likely underpinned by neurodegeneration of key brain structures important for stress and emotional reactivity. Dopaminergic dysfunction that is classically associated with the pathogenesis of PD has been implicated as a mediator due to alterations in structures important for emotional processing (e.g., amygdala) [147]. Indeed, neuroimaging evidence has suggested that PD-induced anxiety is associated with maladaptive activation of the amygdala and functional connectivity to brain regions that mediate mood and cognition [148]. However, other processes are likely contributors since evidence has shown that dopaminergic replacement therapies commonly used for treatment of motor symptoms have modest anxiolytic effects [149]. PD-induced anxiety has also been suggested to be related to serotonergic and noradrenergic pathway dysfunction (e.g., locus coeruleus) in the brain, which may impair anxiety regulation and reactivity to stress.

Despite a lack of available clinical trials specifically addressing music and PD-induced anxiety, music has been shown to induce anxiolytic effects across various populations [35,150]. Music interventions potently modulate various biological systems involved in stress responses, including emotional and autonomic regulatory systems. Indeed, various brain networks associated with emotional and stress responses (e.g., amygdala, prefrontal cortex, hypothalamus) are affected by listening to music and may impose a blunting of hyperactivity in these regions [14]. Maladaptive overactivity in brain regions mediating emotion (e.g., amygdala) has been well described in the pathology of PD, suggesting a therapeutic potential for music [151]. Further supporting anxiolytic effects of music interventions, other evidence has shown lower neuroendocrine markers of stress (e.g., cortisol and salivary amylase) with music interventions, especially in instances of stressful situations [152]. In the context of PD, salivary and humoral markers of physiological stress (e.g., cortisol) have been observed to be elevated in people with PD compared to healthy controls and are associated with worsened functional and behavioral outcomes [153]. Modulation of neuroendocrine stress mediators may induce systemic consequences beneficial to mitigating anxiety and stress responses such as lowering blood pressure and cardiac rate [154]. In alignment with this, music interventions have been shown to modulate autonomic mediators of stress and anxiety. Meta-analytic reviews have suggested moderate effects of music interventions on lowering heart rate and blood pressure and increasing heart rate variability, suggestive of improved autonomic balance and lower physiological stress [53,155,156]. Psychologically, music interventions may also serve to increase dissociation and distract from stressors and perceptions of anxiety [157]. This is likely mediated through attentional shifts and improvements in mindfulness [157,158]. Given that PD-associated stress has been linked to increased rumination of negative thoughts and reduced mindfulness, music interventions may aid in combating psychological and behavioral factors contributing to anxiety [159]. Thus, potential benefits of music interventions on PD-induced anxiety and stress responses are likely mediated by a combination of psychological and physiological effects requiring further inquiry and control trials to reveal therapeutic potential and application.

### 4.8. Pain and Sensory Dysfunction

Chronic pain and sensory dysfunction are primary contributors to the non-motor symptomology of PD, which contribute to lower quality of life and increased incidence of disability. While the prevalence of PD-induced chronic pain is difficult to discern due to co-existing NMSs, recent epidemiological data has suggested that approximately 66% of people with PD experience some form of chronic pain, with females reporting higher incidence and severity than their male counterparts [160]. Phenotypically, PD-induced chronic pain is multifactorial and heterogeneous, including musculoskeletal, neuropathic, and central pain types, with some people with PD experiencing multiple types concomitantly [161,162]. Although chronic pain and sensory dysregulation in PD have been debated as a consequence secondary to aging and other comorbidities, there is clear evidence showing that people with PD experience alterations in pain thresholds and sensitivity compared to healthy counterparts, suggesting alterations in pain as a core manifestation of disease progression [163,164]. Further supporting this, dopaminergic replacement therapies have been shown to partially improve chronic pain symptoms, suggesting that dopamine dysfunction occurring from PD is likely an underpinning mechanism [164]. Neurodegeneration of various brain regions has been implicated in PD-induced chronic pain, including the basal ganglia, nucleus accumbens, anterior cingulate cortex, and prefrontal cortex, which contribute to altered sensory, emotional, and cognitive mediators of pain [165]. In addition to dopamine, serotonergic and noradrenergic dysfunction have also been implicated in PD-induced chronic pain through alterations in pain sensitivity and perception [166]. Treatments for PD-induced chronic pain are typically symptom-dependent, and while dopaminergic replacement strategies are somewhat efficacious, responses to pharmacological treatment are unreliable, suggesting the need for adjunctive therapeutic options.

Direct clinical evidence for the use of music listening as an adjunct therapy in PD is extremely limited. However, the use of music as a non-pharmacologic therapy to combat pain in clinical populations has considerable mechanistic support. Overall pain scores have been shown to decrease among varying populations with music interventions [167]. Furthermore, pain-induced emotional disturbances and use of analgesic pharmacologic therapies have been shown to be reduced with music interventions [167,168]. Music may act as an external distractor, thereby competing with sensory input and reallocating attentional resources of pain perception [169,170]. As previously mentioned, music interventions have been shown to modulate affective state and reduce anxiety, which have been described to exacerbate pain perception in PD [157]. Music may in turn aid in combating negative emotions and intrusive thoughts induced by pain in people with PD through dissociative effects [168]. Modulation of corticolimbic networks has also been described in previous evidence, which may increase reward and alter pain processing by activating endogenous analgesic systems and alter descending inhibitory pathways [41,152]. Interestingly, pain-modulating effects of music appear to be preference-mediated. Indeed, listening to self-selected or preferred music has been suggested to impart a more potent analgesic effect compared to music not chosen by the individual, suggesting that analgesic effects of music are mediated by music choice [171]. This is consistent with other findings showing that perceptions of exertion in response to physical stressors (e.g., high-intensity exercise) are modulated by music [10,11]. Thus, implementing individualized music listening interventions may be most effective in combatting PD-induced pain, although further clinical testing is needed.

## 5. Therapeutic Application, Limitations, Challenges, and Future Directions

Integrating music interventions as an adjunct therapy to traditional treatment for PD represents a feasible, readily available, and easily personalized approach to improve health and NMSs in people with PD. In particular, the personalization of music interventions in anticipatory or reactionary contexts to NMSs of PD highly supports the therapeutic application of music interventions in a wide variety of scenarios. For example, patients and practitioners may use music to target changes in emotional state, autonomic balance, pain perception, or well-being. Organization of musical playlists by music characteristics may further allow patients and practitioners to readily respond in real time or prescribe therapeutic music regimens to facilitate optimal patient responses to combat NMS. Since music interventions are easily customizable, providing patients with control over musical choice may foster a sense of autonomy, which may be particularly meaningful for individuals who have experienced feelings of a loss of control related to disease progression. Therapeutic techniques using a patient’s preferred music during cognitive training, such as lyric completion for speech, may support memory retrieval and sustained attention. Furthermore, the accompaniment of motivational music with physical tasks and exercise may improve the motivational state and physical effort, thus promoting engagement and adherence to physical rehabilitation techniques [10]. This is further supported by a myriad of evidence showing music and auditory cuing as an effective strategy to improve motor processes in people with PD [172,173]. To accomplish this translation of therapeutic practice from evidence, future research needs to standardize study design, forms of PD, symptomology experienced, methods of music selection (i.e., preferred versus non-preferred), and outcomes measured to previously established evidence to allow for direct comparison, enabling greater confidence in findings and comparisons.

Despite the growing mechanistic and behavioral evidence supporting the potential utility of music interventions for NMSs in PD, several challenges continue to limit the translation of this approach into clinical practice. One critical consideration is the incomplete inclusion of NMSs commonly associated with PD. Indeed, there is a wide range of NMSs in PD not currently included, such as gastrointestinal issues and sexual dysfunction, which contribute to disease symptomology and diminished quality of life [174,175]. At this time, many of these NMSs do not have enough evidence or mechanistic support to ascertain whether music interventions may be effective in providing therapeutic benefit. However, given the multi-faceted benefits that music interventions offer, it is plausible that the implementation of targeted music interventions may aid in combating these NMSs, but further research is needed in order to characterize the potential benefits of music interventions across all NMSs associated with PD. Moreover, it is worth noting that the existence of symptom domains in PD is concomitant, whereby motor and NMSs may exist simultaneously and together result in the overarching disease phenotype [176]. For example, increases in experienced pain, a common NMS of PD, may affect or worsen motor outcomes such as gait disturbances due to movement barriers [177]. Therefore, while the current narrative review is limited in scope towards particular NMSs of PD that have neuromechanistic support from the music literature at this time, both motor and NMSs should be considered an integrated continuum that needs to be accounted for in their totality as synergistic components to overall symptomology in PD.

Furthermore, the inherent characteristics of music are highly variable across studies and individuals. Musical tempo, rhythm, harmonic structure, familiarity, lyrical content, and personal preference all influence physiological and psychological responses to music interventions [10,11,30,178,179]. Evidence across populations suggests that self-selected or preferred music produces stronger dopaminergic, emotional, and motivational effects than externally prescribed selections. Conversely, music interventions that are mismatched to an individual’s arousal state, emotional needs, or disease stage may induce negative or even maladaptive responses, including increased sympathetic activation or emotional distress. Thus, future studies connecting music characteristics and curating personalized music plans to an individual’s unique symptom presentation are needed to maximize the benefits of music interventions to achieve therapeutic goals.

A second major limitation is the overreliance on evidence derived from healthy individuals or non-PD clinical populations. While mechanistic studies in healthy adults provide critical insight into how music modulates neural, autonomic, and affective systems, PD is characterized by progressive neurodegeneration across dopaminergic, serotonergic, noradrenergic, and cholinergic systems. As a result, music-induced responses observed in healthy populations may not translate directly to individuals with PD, particularly in later disease stages or in those with cognitive impairment. Additionally, heterogeneity in disease phenotype, medication state (“on” vs. “off”), and comorbid neuropsychiatric symptoms further complicates interpretation. Future research should prioritize PD-specific investigations that stratify participants by disease stage, cognitive status, and dominant NMS profile, allowing for clearer identification of who is most likely to benefit from music-based interventions.

Another unresolved issue concerns the distinction between acute and chronic responses to music. There is an abundance of evidence demonstrating short-term improvements in mood, stress, arousal, and/or pain perception during or shortly after music exposure. However, there is a paucity of evidence to suggest whether these effects persist with repeated use or translate into long-term symptom modification. Consistent music exposure may promote sustained neuroplastic changes, improved autonomic regulation, and behavioral habit formation, but optimal “dosing” parameters such as frequency, duration, timing, and progression remain unclear. Longitudinal randomized trials are needed to determine whether chronic implementation of music interventions can meaningfully alter the trajectory of NMS progression, reduce symptom burden over time, or enhance responsiveness to existing pharmacologic and rehabilitative therapies.

Finally, further distinction must be made between passive music listening and active music therapy. Currently, there is not enough available literature differentiating between passive and active music interventions on NMSs in PD to allow for comprehensive therapeutic recommendations or insight. It is important to note that music interventions have a wide degree of heterogeneity in the biological systems affected between different modes of therapy (i.e., passive listening versus singing or playing an instrument). Since not all modes of music delivery can be distinguished currently, application of the synthesis in this narrative review should be treated with caution until distinct advantages between therapeutic modalities can be ascertained. Comparatively, passive listening is highly accessible, scalable, and feasible for home-based implementation, making it an attractive adjunct for individuals with mobility limitations or advanced disease. Active music therapy may offer additional cognitive, motor, and social benefits but requires trained therapists and structured delivery. While both approaches hold tremendous value for therapeutic potential, determining when passive versus active approaches are most appropriate, and whether hybrid models yield additive benefits, represents a key future direction. Collectively, addressing these challenges through rigorously designed, PD-specific studies will be essential for refining music-based interventions and advancing their role as a personalized, non-invasive strategy for managing NMSs in PD.

## Figures and Tables

**Table 1:** Search terms used to search for the included literature for the narrative review.

Category	MeSH Terms	Other Keywords
Population	Parkinson’s disease	Dementia, Neurodegeneration, Non-motor, Non-motor symptoms, Parkinson’s, Parkinsonism
Intervention	Music, Music therapy	Auditory, Music listening
Measurement	Functional Neuroimaging, Magnetic Resonance Imaging Neuroimaging, Neuropsychological Tests, Positron-Emission Tomography, Psychophysiological	Functional MRI, Physical performance, Physiological, Psychological, Subjective, Task performance
Psychological Variables	Affect, Anxiety, Attention, Apathy, Arousal, Cognition, Cognitive dysfunction, Depression, Emotion, Executive function, Mood disorder, Motivation, Quality of life, Subjective stress	Affective state, Dissociation, Encoding, Enjoyment, Language, Long-term memory, Memory, Short-term memory, Vigor
Physiological Variables	Amygdala, Blood pressure, Brain, Chronic pain, Cortisol, Dopamine, Dysautonomia, Fatigue, Heart rate, Hyperalgesia, Norepinephrine, Nucleus Accumbens, Pain, Serotonin, Sleep	Anterior Cingulate Cortex, Autonomic, Cortical, Heart rate variability, Limbic, Mesocorticolimbic, Paralimbic, Parasympathetic, Physiological Stress, Prefrontal cortex, Sympathetic

## Data Availability

No new data were created or analyzed in this study.
